# Anorexia Nervosa Dampens Subjective and Facial Pain Responsiveness

**DOI:** 10.3390/brainsci15101082

**Published:** 2025-10-07

**Authors:** Stefan Lautenbacher, Miriam Kunz, Karl-Jürgen Bär

**Affiliations:** 1 Medical Psychology and Sociology, Medical Faculty, University of Augsburg, 86135 Augsburg, Germany; miriam.kunz@med.uni-augsburg.de; 2 BamLiD, Bamberger Living Lab Dementia, University of Bamberg, 96045 Bamberg, Germany; 3 Department of Psychosomatic Medicine and Psychotherapy, University Hospital Jena, 07740 Jena, Germany; karl-juergen.baer@med.uni-jena.de

**Keywords:** anorexia nervosa, pain threshold, facial expression, pain communication

## Abstract

**Background/Objectives:** Individuals with anorexia nervosa (AN) are known to exhibit both reduced pain sensitivity—when assessed via thresholds and subjective ratings—and diminished facial expressions of emotion. Therefore, investigating the facial response to pain in this population is of particular interest. **Method:** Seventeen patients with AN and 18 age- and sex-matched healthy controls were assessed using a thermode to induce heat pain. Subjective pain measures included pain threshold, pain tolerance, and pain ratings of supra-threshold stimuli, rated on a numerical rating scale (NRS). Facial responses to the suprathreshold stimuli were analyzed using the Facial Action Coding System (FACS). Eating pathology was assessed using the Eating Attitudes Test (EAT-26), the Eating Disorder Inventory-2 (EDI-2) and the body mass index (BMI), while depression was measured using the Beck Depression Inventory-II (BDI-II). **Results:** Compared with healthy controls, AN patients showed altogether significantly reduced facial expressions of pain, with particularly pronounced reductions in Action Units AU 6_7 and AU 9_10. In contrast, subjective pain measures showed only marginal differences between groups. Importantly, the reduction in facial expression could not be accounted for by differences in pain thresholds or ratings, nor by levels of eating pathology or depression. **Conclusions:** Individuals with AN display a markedly reduced facial expression of pain, which was observed for the first time, consistent with similar findings regarding the facial expressions of emotions. As this reduction cannot be explained by subjective pain report, it suggests that the communication of pain is impaired on two levels in AN: both in verbal and in nonverbal signaling. This may hinder the ability of others to recognize and respond to their pain appropriately.

## 1. Introduction

Anorexia nervosa (AN) is an eating disorder characterized by restriction of food intake leading to low body weight, typically accompanied by intense fear of weight gain and a disturbed body image [1]. The neurobiological consequences of extreme caloric restriction are manifold, and it is often difficult to distinguish between alterations caused by the disorder itself and those resulting from starvation [2]. Among the many dysfunctions, a reduction in pain sensitivity has repeatedly been observed in acutely ill patients but not in those who have recovered following weight restoration [3,4,5,6,7,8]. Furthermore, food restriction alone does not seem to play a substantial role as individuals without an eating disorder, who engage in caloric restriction for scientific or cosmetic reasons, show no changes in pain sensitivity [9]. Interestingly, peripheral skin temperature [5,6] as well as depression [10,11] seem to be of relevance, given that significant correlations between these variables and pain thresholds have been reported in AN patients. No clear-cut relationship between eating pathology and pain sensitivity could be demonstrated, with a trend of lower pain sensitivity in anorectic patients with additional bulimic symptoms [6]. Changes in pain induced brain activation, especially in the cingulate and insular pain-specific cortex regions, have also occasionally been found [12,13]. In sum, the reduction in pain sensitivity in the acute phase of the disorder is a robust finding obtained in several studies using various experimental pain stimuli, but the underlying causal mechanisms remain unclear.

The reduction in pain sensitivity in patients with AN increases the risk of being affected by noxious events and may also hinder their ability to verbally communicate pain, limiting opportunities for others to provide support or intervene. This raises the question of whether individuals with AN utilize alternative forms of pain communication, such as non-verbal cues. Facial responses—commonly associated with and indicative of pain—appear to be a particularly relevant alternative. However, it is important to note that facial responses to painful stimuli are often only weakly correlated with self-reported pain [14]. This may be due to the fact that—after the common nociceptive encoding in the brain—pain ratings rely on cognitive and linguistic cortical areas (dorsolateral prefrontal areas, Broca’s area) whereas facial expressions of pain are more reflexive and ultimately modulated by inhibitory prefrontal motor gates (medial, ventrolateral prefrontal cortex) [15,16]. Due to these distinct pathways involved in the final stages of behavioral pain expression, discrepancies between subjective pain ratings and facial expressions are to be expected.

In any case, it is worth investigating the facial activity during pain in AN and compare it with subjective pain ratings in an experimentally well-controlled paradigm. The background knowledge from the literature informed us, however, that persons with AN are less facially expressive when positive and negative emotions were considered [17,18,19,20,21]. Thus, it is unlikely that they use nonverbal facial responses to pain more intensively to balance out the reduced verbal response to pain. Therefore, our summary hypothesis was that individuals with anorexia nervosa subjectively experience less pain (reflected in reduced pain ratings and increased pain threshold) and display less facial pain behavior (indicated by less intense and frequent Action Units, as coded by the Facial Action Coding System (FACS) [22]) independently of, and maybe even exceeding the reduction in the subjective experience, than matched healthy controls. This assumption was tested for the first time.

## 2. Materials and Methods

### 2.1. Participants

17 patients with anorexia nervosa (AN) and 18 age- and sex-matched controls participated in the study. We based our sample size calculations (Sample Power 2.0, SPSS Inc., Chicago, IL, USA) on a previous study of our group where we investigated pain responses in AN [5] and in depression [23], which reported a large effect size (Cohen’s d = 1; two-tailed; power = 0.8). AN patients were all inpatients at the Department of Psychiatry and Psychotherapy of the University hospital Jena. Patients were diagnosed by a staff psychiatrist. They fulfilled DSM-IV criteria (Diagnostic and Statistical Manual of mental disorders, 4th edition, published by the American Psychiatric Association) for AN according to the Structured Clinical Interview for DSM-IV Axis I disorders (SCID-I [24]). Control participants were recruited amongst medical students and staff by advertisements posted in the university hospital buildings. Demographic data of the participants are presented in Table 1. With exception of the educational background (patients had less years of education), demographic data did not differ between patients and control participants. As expected, AN patients had a significantly reduced BMI compared to healthy controls (see Table 1).

To assess the severity of eating disorder in individuals with AN, we used the Eating Attitude Test-26 (EAT-26 [25,26]) and the Eating Disorder Inventory-2 (EDI-2 [27]). Both are validated self-report questionnaires widely used to identify abnormal eating patterns.

We also assessed depressive symptoms in all participants using the Beck Depression Inventory (BDI-II [28]). As can be seen in Table 1, AN patients scored significantly higher on the Beck Depression Inventory.

This study was carried out in accordance with the Declaration of Helsinki. After having been informed about the nature of intended procedures, all participants gave written informed consent to a protocol approved by the Ethics Committee of the University Hospital Jena.

*t*-tests (age, BMI, BDI) and χ^2^ Tests (gender, education, smoking, coffee consumption, sport) were conducted to look for differences between groups, results are listed in the right column.

### 2.2. Materials and Procedure

**Figure 1:**
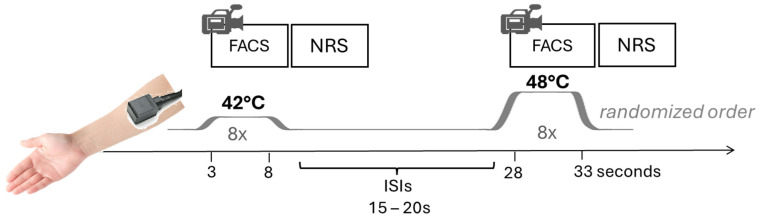
Heat stimulation protocol. Participants received non-painful (42 °C) and painful (48 °C) stimuli in a pseudo-randomized order, during which facial expressions of pain (FACS) were recorded. Following each stimulus participants provided self-report ratings (NRS).

Prior to the experiment, participants were thoroughly familiarized with the procedures. The session lasted approximately one hour, during which participants sat upright in a comfortable armchair. The experimenter remained behind a computer screen to prevent eye contact, and social interaction during pain testing was minimized, limited to prompting participants to rate the stimuli. The session included completion of questionnaires, assessment of pain sensitivity (pain threshold), and measurement of facial and subjective responses to moderately painful heat stimuli. Pain was induced using a Peltier-based, computerized thermal stimulator with a 3 × 3 cm^2^ contact probe (Medoc TSA-2001; Medoc Ltd., Ramat Yishai, Israel), which was applied to the left forearm (see Figure 1).

### 2.3. Assessment of Pain Thresholds and Pain Tolerance Thresholds

To determine participants’ heat pain thresholds and heat pain tolerance, we employed the method of limits. Starting from a baseline of 35 °C, temperature increased at a rate of 0.5 °C/s. Participants were instructed to press a stop button as soon as the sensation became slightly painful (pain threshold) or intolerable (pain tolerance), respectively. Pressing the stop button caused the temperature to return to baseline (35 °C) at a rate of 10 °C/s. Following instruction, five trials were conducted for pain threshold assessment, followed by five trials for pain tolerance assessment. Inter-stimulus intervals (ISIs) ranged from 8 to 12 s. The mean of the five trials was calculated to provide an estimate of each participant’s pain threshold and pain tolerance.

### 2.4. Assessment of Subjective and Facial Responses to Painful Heat Stimuli

Following threshold assessments, eight non-painful and eight painful thermal stimuli of fixed intensities (see Figure 1) were applied in the same pseudo-random order for all participants. The alternation of stimulus intensities should keep vigilance high. The selection of stimulation temperatures was guided by prior empirical evidence. Specifically, 42 °C was applied as a reliably non-painful stimulus and 48 °C as a reliably painful stimulus. These values were derived from previous studies conducted by our group in both healthy individuals and patients with major depressive disorder (MDD) [23,29,30,31], demonstrating that the chosen intensities are consistently perceived as non-painful and painful, respectively, by the majority of participants, while the painful stimulus remains below the individual pain tolerance threshold. The non-painful heat intensity served as a reference to assess baseline facial expression unrelated to pain. The temperature increased from baseline (38 °C) at a rate of rise 4 °C/s to the pre-set temperatures, remained at a plateau for 5 s, and returned to baseline at a rate of temperature change of 4 °C/s. Long ISIs were used to prevent sensitization (15–20 s) and allow enough time for rating the stimulus intensity.

Self-report ratings: Participants evaluated the thermal stimuli using a two-step procedure to achieve sufficient resolution across the full range from non-painful to painful temperatures [23]. In the first step, participants were asked to indicate—after each stimulus—whether the stimulus received was painful or not. In a second step, participants then verbally rated the stimulus either on a numerical ratings scale (NRS) ranging from either 0 (‘no heat’) to 10 (‘extremely warm’) in case the stimulus was rated as non-painful; or on a separate scale ranging from 0 (‘no pain’) to 10 (‘extremely strong pain’) in case the stimulus was rated as painful. All painful stimuli (48 °C) were rated as painful.

Facial expression of pain: The face of the participant was videotaped throughout the thermal stimulation. The camera was placed in front of the participant in a distance of approximately 2 m. The camera was positioned approximately 2 m in front of the participant. Before each stimulus, participants were instructed to focus on a target (‘x’) on the wall behind the camera to ensure a frontal view and to refrain from speaking. A LED, visible to the camera but not to participants, was illuminated during the 5 s stimulus plateau to mark stimulus onset and offset.

Facial responses were quantified using the Facial Action Coding System (FACS [22]), a fine-grained anatomically based system that is considered the gold standard when assessing facial expressions, including the facial expression of pain [32]. The FACS distinguishes 44 different action units (AUs) produced by a single muscle or a combination of muscles. A certified FACS coder scored AU frequency and intensity (5-point scale) using Observer Video-Pro 14 software (Noldus Information Technology). Scoring focused on 5 s segments starting at the target temperature, with 16 segments per participant (8 non-painful, 8 painful). AUs representing the same muscle (AU1_2, AU6_7, AU9_10, AU25_26_27) were combined [23]. FACS coding was conducted in another center (University of Bamberg) and was blind to the diagnostic status of the participant (AN or healthy control).

Pain-relevant AUs were selected if they occurred in >5% of painful segments and were more frequent during painful (48 °C) than non-painful (42 °C) stimulation (d ≥ 0.5) [33,34]. Only AUs pain-indicative in both groups were used for group comparisons (shaded in Table 2). AU intensity and frequency were multiplied and averaged to create a composite facial pain score, which was square-root-transformed (composite score_sqrt_) as performed in previous studies [23,35], given its left-skewed distribution Significant composite results were followed by analyses of individual pain-indicative AUs using the same preprocessing.

### 2.5. Statistical Analysis

Psychophysical data: To compare threshold estimates between groups a repeated measurement ANOVA is conducted (within subject factor: threshold type—pain threshold, tolerance threshold). Self-report ratings of pain (only to the 48 °C stimuli) are compared between groups using an ANOVA.

Facial responses: Facial expressions of pain (composite score of facial expressions to the 48 °C stimuli) are compared between groups using an ANOVA. In case of a significant group difference, a MANOVA will be conducted by entering the single pain-indicative AUs as dependent variables; to investigate whether group changes are consistent across AUs.

Modulating factors/co-variates: We will additionally assess how depression (BDI (correlation analysis)) and severity of AN (BMI, EDI, EAT-26) (regression analysis) might impact pain outcomes. We will limit these analyses to those pain outcomes that differ between groups.

All tests were conducted using SPSS 24. Findings were considered to be statistically significant at α < 0.05.

## 3. Results

### 3.1. Psychophysical Data/Subjective Responses to Pain

**Figure 2:**
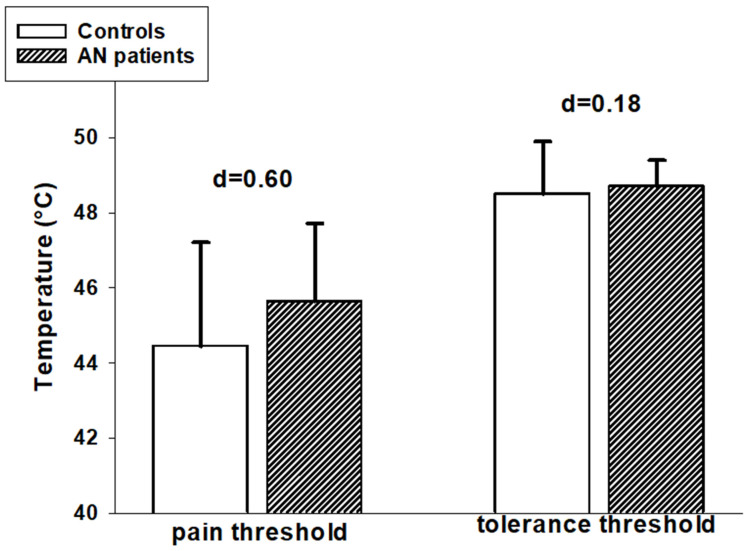
Mean values (+SD) of pain threshold and pain tolerance thresholds in AN patients and in healthy controls. Effect sizes (Cohen’s d) for group comparisons are displayed.

Pain and tolerance thresholds: As expected, the temperature characterizing pain tolerance thresholds was significantly higher compared to the temperature of pain thresholds (F(1,33) = 80.78, *p* < 0.001) (see also Figure 2). When comparing pain thresholds and pain tolerance thresholds between groups we did not find a significant overall group effect (F(1,33) = 2.60, *p* = 0.116). However, there was a trend towards a significant interaction effect between “group” and “threshold type” (F(1,33) = 2.98, *p* = 0.094). As can be seen in Figure 2, the temperature difference between pain thresholds and pain tolerance thresholds was reduced in AN patients. As computation of effect-sizes (Cohen’s d) showed, this was due to pain threshold levels tending to be increased in AN patients compared to controls (d = 0.60, medium effect size), whereas pain tolerance levels were more comparable between groups (d = 0.18, no effect).

**Figure 3:**
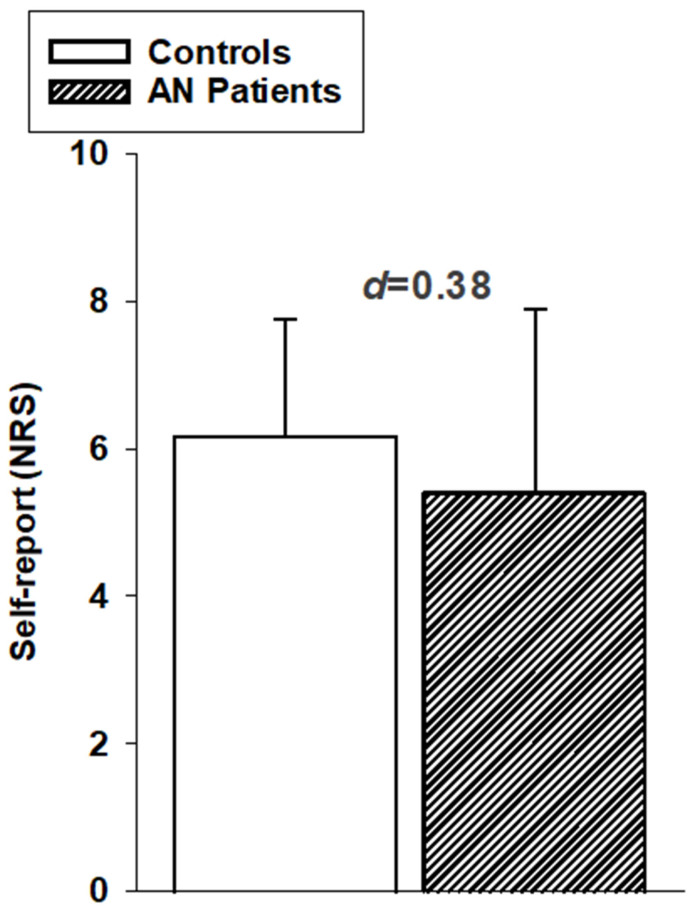
Numerical self-report ratings (mean values (+SD)) to painful heat stimulation (48 °C). Ratings are given separately for AN patients and healthy controls. Effect sizes (Cohen’s d) for group comparisons are displayed.

Subjective responses to painful heat stimulation: We were successful in eliciting painful sensations by applying 48 °C stimuli. In our two-step rating approach, all participants indicated that the stimulus was painful in the first step and then provided a rating between 0 (no pain) and 10 (extremely strong pain). We did not find any significant group differences in pain ratings (F1,34) = 1.23, *p* = 0.275; d = 0.38, small effect size). Although descriptive data displayed in Figure 3 suggest that AN patients rated the painful heat stimuli as slightly less intense, this difference was not significant.

Thus, the descriptive data shown in Figure 2 and Figure 3 suggest a trend toward slightly reduced pain sensitivity in patients with AN, although this difference did not reach statistical significance.

### 3.2. Facial Expression of Pain to Painful Heat Stimulation

**Figure 4:**
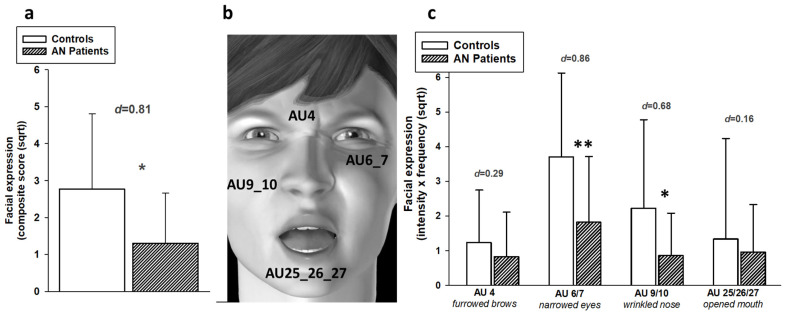
Pain-indicative facial responses to painful heat stimulation (48 °C) in AN patients and healthy controls (mean values; +SD). (**a**) composite score_sqrt_ of facial expressions of pain; (**b**) illustration of the pain-indicative AUs; (**c**) single pain-indicative AUs (AU4sqrt, AU 6_7sqrt, AU 9_10sqrt, AU25/26/27sqrt). Effect sizes (Cohen’s d) for group comparisons are displayed, ** *p* < 0.01, * *p* < 0.05.

Table 2 provides an overview of the individual facial Action Units (AUs) observed during painful heat stimulation (48 °C). There was substantial overlap in the types of AUs displayed by AN patients and healthy controls. The AUs identified as pain-indicative (AU4, AU6_7, AU9_10, AU25_26_27) are consistent with previous research on facial expressions of pain [36] and are illustrated in Figure 4b. As described above in the Method Section, these pain-indicative AUs were combined to form a composite score of facial expressions of pain.

The composite score_sqrt_ of facial expressions of pain significantly differed between groups (F(1,34) = 6.27, *p* = 0.017; d = 0.81, strong effect size). As can be seen in Figure 4a, AN patients showed markedly reduced facial expressions of pain in response to painful heat stimulation compared to healthy controls.

To investigate whether this reduced facial display of pain in AN patients occurs consistently across the different pain-indicative AUs, the four pain indicative AUs (see Figure 4b) were compared between groups using a multivariate analysis of variance. The multivariate outcome replicated the significant group effect for facial responses to pain (F(4,31) = 2.75, *p* = 0.046). As univariate outcomes showed, this significant group effect was mainly due to AN patients responding with decreased narrowing of the eyes (AU6_7; *p* = 0.012, d = 0.86, strong effect) and decreased wrinkling of the nose (AU9_10; *p* = 0.039, d = 0.72, moderate effect) in response to the painful stimulation compared to healthy controls. In contrast, contraction of the eyebrows (AU4; *p* = 0.374, d = 0.30, small effect) and opening of the mouth (AU25_26_27; *p* = 0.321, d = 0.34, small effect) did not differ between groups. Thus, although Figure 4c shows that, descriptively, all pain-indicative AUs were less vigorously displayed by AN patients, the reduction was particularly pronounced in the eye and nose regions. Clinically, this attenuation may lead to underestimation of patients’ pain by caregivers.

Overall, AN patients showed slightly (but not significantly) reduced subjective sensitivity to thermal heat pain (increased pain threshold and reduced pain ratings). With regard to facial responses, we found significant group differences in moderate to strong effect sizes, with AN patients showing reduced facial expressions of pain in response to painful heat stimulation. To investigate whether the reduced facial responses to pain in AN patients was simply due to the tentatively reduced subjective pain experience (i.e., AN patients express less pain facially because of the subjective experience of less pain), we conducted a covariate analysis (with composite score_sqrt_ of facial expressions as dependent variable and entering the NRS pain rating as covariate). The significant group difference in facial responses to pain remained, even when controlling for self-report ratings (F (1,33) = 2.31, *p* = 0.024). Thus, the reduced facial responses to pain in AN patients were not simply due to a lower subjective pain experience.

Given that the depression scores varied between groups (see Table 1), we tested whether the reduced facial responses to pain in AN patients can be explained by the degree of depression. We did his by correlating the BDI scores with the composite scores_sqrt_ of facial responses to pain and found no significant correlation (r = −0.26, *p* = 0.132).

Furthermore, we were interested whether the symptomatology of AN as indicated by the BMI, EAT and EDI scores (see Table 1) might impact the facial responsiveness to pain. To test this, a regression analysis was conducted with BMI, EAT and EDI scores as predictors and the composite scores_sqrt_ of facial responses to pain as criterion variable (we tested for multicollinearity and found no violation (VIF < 5; tolerance > 0.2)). The regression analysis was only conducted within the group of AN patients). Regression analysis assumptions were met. The symptomatology of AN was unrelated to the facial responsiveness to pain (r^2^ = 0.036, *p* = 0.919).

## 4. Discussion

We replicated the frequently reported reduction in subjective pain responsiveness among individuals with anorexia nervosa (AN) only as a trend, with small to medium, non-significant effects for pain threshold, pain tolerance, and pain ratings. This slight deviation from the existing literature will be discussed further below. In contrast, facial responses to pain in AN patients were significantly reduced; a finding that could not be explained by subjective pain ratings when included as a covariate and thus represents an independent change that goes beyond the reduction in pain sensitivity in persons with AN.

There are many examples in the literature noting that facial expressions of emotions are dampened in patients with anorexia nervosa, consistent with the reduced facial expression of pain observed in the present study [17,18,19,20,21]. This relates to a longstanding discussion whether pain can be equated with emotions, at least with certain negative emotions, which could not yet be settled [37]. Pain clearly comprises more components than the emotional one alone (e.g., also sensory, cognitive, behavioral components). In an earlier study [38], we could demonstrate using hypnotic suggestions that certain facial movements (AU4, AU9_10) during pain are enhanced when participants focused on the unpleasantness of the pain experience, whereas others (especially AU6_7) were more prominent in a more sensory-focused mindset. In the present study, individuals with AN displayed less AU6_7 and AU9_10 in response to pain. Thus, the anorectic participants exhibited a reduced facial display of both the affective and sensory dimensions of pain; however, the diagnostic value of this finding may be limited compared to more prominent clinical symptoms of AN. In total, the present findings are consistent with the phenomenon of reduced facial expression of emotions in AN, suggesting a potential overlap that warrants further empirical investigation. The altered facial expressions of emotions in anorexia nervosa may be explained by a disturbance of emotion regulation, such as altered distress tolerance, emotion avoidance or emotion inhibition [18,39,40]. It is plausible that similar anorexia-specific ways of emotion-regulation strategies underlie the dampened facial expression of pain. However, another explanation may be that facial responses depend on intact muscle function, which may be impaired, as both neuropathy and myopathy can result from prolonged malnutrition and nutritional deficiencies associated with anorexia nervosa [41,42]. In a review, individuals with anorexia nervosa were reported to exhibit 16% to 70% lower muscle strength compared to healthy controls or age-matched normative values, with an average reduction of 35.2% [43]. To test this hypothesis of neuromuscular weakness, patients with anorexia nervosa could be trained to intentionally reproduce specific Action Units—such as AU4 (brow lowerer), AU6 (cheek raiser), and AU9 (nose wrinkle)—to enable direct comparison with healthy control subjects. This approach could assess not only the visibility of facial movements but also the underlying motor function, using surface EMG recordings of the corresponding muscles. EMG provides a sensitive measure for detecting potential neuromuscular weaknesses.

We assessed questionnaires targeting the eating symptomatology (EAT, EDI) and the level of depression (BDI) and determined body weight (BMI). None of these variables showed any significant relationship with the facial expression of pain. Thus, we could not corroborate findings suggesting significant correlations of reduced facial expression of positive and negative emotions with eating pathology and depression [18]. This may be a slight hint that the facial expression of emotions differ from those for pain as regards the underlying mechanism.

The question remains why we could not replicate the positive findings of other authors regarding group differences between acutely ill individuals with anorexia nervosa and healthy participants [4,5,6,7,8]. We clearly missed the significance level of group differences, with small effect sizes of d = 0.18 and d = 0.38 as regards pain tolerance and pain ratings. However, the only consistently robust findings in the literature on individuals with AN have been those regarding elevated pain thresholds. Here, we obtained an effect size of d = 0.60, which can be classified as medium effect and would pass the significance threshold of alpha = 0.05 already when investigating a sample of 36 participants. Pain threshold differences in AN reported in previous studies were often of moderate to strong effect sizes [4,5,6,7,8]. Thus, we essentially observed the same phenomenon reported in earlier studies, although at the lower end of the effect size range. However, we did not reach statistical significance, likely due to our small sample size. As stated in the Method Section, we based our sample size calculations on a previous study that we conducted in patients with depression using a similar pain protocol.

Given the slightly reduced subjective experience of pain and the markedly diminished facial responses, all essential channels of acute pain communication appeared to be limited in persons with AN. Furthermore, the communication of pain might not only be reduced in individuals with AN but might also be ambiguous. Given that the reduction in the facial responses to pain was not correlated with the subjective pain report, some patients may facially signal less pain but report normal pain levels and the other way round. Thus, underreporting and misreporting of pain and other aversive sensations may be more the rule than the exception. This issue may also be characterized by terms such as alexithymia or disturbed interoception. Carers, relatives and friends should be aware that individuals with anorexia nervosa are encapsulated as regards their internal experience of pain and that accessing this private domain of aversive experience may require sustained effort, time, and considerable empathy. It should be kept in mind that patients with anorexia nervosa, often plagued by feelings of guilt [44], may intentionally engage in self-injury. In such cases, others may be uncertain about how best to respond and provide care, particularly when pain signals are unclear.

There are several limitations that ought to be mentioned. First, the FACS coding can and should be conducted in a blinded fashion so that the coder does not know which group the person belongs to (e.g., patients vs. healthy participants). However, weight loss alters facial physiognomy, potentially revealing group status. As only the face was videotaped, this may have been less apparent in some participants. Moreover, since the coding rules provided by the FACS are quite objective and reliable, biasing effects of this limitation can be expected to be small. Finally, the individual conducting the FACS coding was kept blind to the study’s hypotheses. However, systematic alterations in FACS coding due to emaciation cannot be ruled out and should be addressed in future research. Second, our sample of anorectic patients was notably limited. This constraint stemmed from both a restricted recruitment pool and the substantial time investment required for FACS coding, which significantly hindered our ability to expand the sample further.

## 5. Conclusions

In sum, we demonstrate a reduced facial expression of pain in individuals with AN, which could not be explained by subjective pain ratings as covariate; and thus, represents an independent change that goes beyond the reduction in pain sensitivity in persons with AN. The reduced facial signaling of pain was due to a lesser display of AUs 6_7 (narrowed eyes) and 9_10 (wrinkled nose), which are very common as pain indicative facial responses [36]. Carers, relatives and friends, who might try to decode this reduced facial pain signal, might be further confused because there is no clear relationship to the verbal report of pain. Since similar changes have been reported for the facial expression of emotions, it remains to be demonstrated whether our findings were specific for pain or general to many internal states, at least to the aversive ones. Nevertheless, pain perception is not currently a central factor in the diagnosis or management of anorexia nervosa, but it may offer valuable insights into the neurobiological underpinnings of the disorder.

## Figures and Tables

**Table 1:** Clinical and demographic data of participants.

Parameter	Healthy Controls	AN Patients	*p*
Number of participants (N)	18	17	
Male/female (N)	0/18	0/17	
Age in years (mean (SD))	25.6 (5.3)	27.3 (8.9)	0.46
BMI (mean (SD))	22.1 (2.5)	15.3 (1.7)	**<0.001**
*Education*			
10 years at school	1	7	**0.012**
12 years at school (A-Level)	17	10	
Smoker/non-smoker (N)	3/15	5/12	0.369
Coffee consumption yes/no (N)	9/9	13/5	0.086
Sport (yes/no) (N)	10/8	10/7	0.845
*Depression scales*			
BDI (mean (SD))	3.1 (2.1)	23.2 (8.5)	**<0.001**
*Eating Disorder scales*			
EDI (mean (SD))	-	3.6 (6.8)	
EAT 26 (mean (SD))	-	1.3 (0.6)	
*Pain outcomes* Pain threshold (mean (SD))	44.0 (3.3)	45.6 (2.1)	
Tolerance threshold (mean (SD))	48.5 (1.3)	48.7 (0.7)	
Self-report, NRS (mean (SD))	6.2 (1.6)	5.5 (2.4)	

SD—standard deviation; BMI—Body Mass Index; BDI—Beck’s Depression Inventory; EDI—Eating Disorder Inventory; EAT 26—The Eating Attitude Test. Sub-headings are written in italics. Significant group differences are marked in bold.

**Table 2:** Facial Action Units (AUs) (i) occurring in more than 5% of the painful segments and (ii) considerably more during pain (48 °C) compared to non-painful (42 °C) segments (effect sizes (d) > 0.5). Values are given separately for healthy controls and AN patients.

		Healthy Controls		AN Patients	
		Frequency	d	Frequency	d
AU 1/2	raised eyebrows	9%	0.33	-	-
* AU 4	furrowed brows	18%	**0.97**	12%	**0.54**
* AU 6/7	narrowed eyes	86%	**1.51**	37%	**1.21**
* AU 9/10	wrinkled nose	43%	**1.35**	11%	**0.63**
AU 14	dimpler	14%	0.13	7%	0.21
AU 17	chin raiser	7%	0.34	-	**-**
* AU 25/26/27	opened mouth	39%	**0.51**	15%	**1.53**
AU 43	closed eyes	-	-	6%	0.30

* Action Units selected for further analyses because they occurred in >5% of all painful segments and occurred more during painful segments compared to non-painful segments (effect size > 0.5). These selected AUs are marked in bold.

## Data Availability

The data presented in this study are available on request from the corresponding author. Due to the inclusion of sensitive patient information, including video recordings, the datasets cannot be shared openly in order to protect patient privacy and comply with ethical requirements.

## Abbreviations

The following abbreviations are used in this manuscript:

AN	Anorexia Nervosa
AUs	Action Units
BDI-II	Beck Depression Inventory-II
BMI	body mass index
EAT-26	Eating Attitudes Test
EDI-2	Eating Disorder Inventory-2
FACS	Facial Action Coding System
MDD	Major Depressive Disorder
NRS	Numerical Rating Scale
sqrt	Square root
